# Two-Week versus Six-Month Sampling Interval in a Short-Term Natural History Study of Oral HPV Infection in an HIV-Positive Cohort

**DOI:** 10.1371/journal.pone.0011918

**Published:** 2010-07-30

**Authors:** Carole Fakhry, Elizabeth Sugar, Gypsyamber D'Souza, Maura Gillison

**Affiliations:** 1 Department of Otolaryngology-Head and Neck Surgery, Johns Hopkins Hospital, Baltimore, Maryland, United States of America; 2 Department of Biostatistics, Johns Hopkins Bloomberg School of Public Health, Baltimore, Maryland, United States of America; 3 Department of Epidemiology, Johns Hopkins Bloomberg School of Public Health, Baltimore, Maryland, United States of America; 4 The Ohio State University Comprehensive Cancer Center, Columbus, Ohio, United States of America; University of Toronto, Canada

## Abstract

**Background:**

Oral HPV infections detected six-months apart were compared to those detected bi-weekly, in an HIV-positive cohort, during the intervening months to elucidate systematic biases introduced into natural history studies by sampling interval.

**Methods:**

Fourteen consecutive oral rinse samples were collected every two weeks for six months from an HIV-positive cohort (n = 112) and evaluated for the presence of 37 HPV types. The cumulative probability of type-specific HPV detection at visits 1 through 14 was determined as a function of infection categorized at visits 1 and 14 as persistent, newly detected, cleared or absent. Transition models were used to evaluate the effect of HPV viral load (measured by RT-PCR for HPV 16, 18, 31, 33, 35) on infection persistence.

**Results:**

The average point prevalence of oral HPV infection was similar at two-week and six-month sampling intervals (45% vs. 47%, p = 0.52), but cumulative prevalence was higher with the former (82% vs. 53%, p<0.001) as was the cumulative prevalence of type-specific infections (9.3% vs 3.8%, p<0.0001). Type-specific infections persistent under a six-month sampling interval had a high probability (0.93, 95%CI 0.83–0.98) of detection at 50% or more of the intervening visits and infections that were absent had a high probability (0.94, 95% CI 0.93–0.95) of no interval detection. The odds of detection at any visit significantly increased for each unit increase in HPV viral load at the previous visit.

**Conclusions:**

Six-month sampling is appropriate to model factors associated with type-specific oral HPV infection persistence but may misclassify HPV-exposed individuals as unexposed.

## Introduction

Oral human papillomavirus (HPV) 16 infection is strongly associated with oropharyngeal squamous cell carcinoma (OPSCC)[1]. Moreover, seropositivity to HPV16 elevates risk for development of OPSCC[2]. Although consistent with a temporal link between oral HPV infection and development of OPSCC, oral HPV natural history studies have not been reported. Such studies will be critical for clarifying potential use of oral HPV detection in OPSCC screening programs.

An important factor for HPV natural history studies is the sampling interval, and six-month sampling intervals are commonly used for cervical studies. We have previously evaluated the feasibility of a six-month sampling interval for oral HPV infection[3]. However, a recent natural history study of cervical HPV infection among adolescent women using a two-week sampling interval revealed short-lived infections (< lasting six-month) were common. As a consequence, the cumulative prevalence was higher in this study relative to prior studies in similar populations[4].

We performed a short-term natural history study of oral HPV infections to compare the results of a two-week to a six-month sampling interval to identify potential systematic biases introduced into natural history studies by the frequency of sampling. Oral HPV infections refers to infections of the oral cavity and oropharynx. An HIV-positive cohort, known to be at elevated risk for oral HPV infection and related malignancies, was selected as our study population.

## Materials and Methods

### Ethics statement

This study was conducted according to the principles expressed in the Declaration of Helsinki. This study was approved by the Institutional Review Board of Johns Hopkins Hospital (application number 03-11-24-04). All patients provided written informed consent for the collection of samples and subsequent analysis.

### Study population

A convenience sample of 112 HIV-positive patients from the Johns Hopkins Hospital was recruited during March 2005. Eligibility criteria included known HIV-positive status and willingness to comply with bi-monthly (every two week) visits for six months. Enrollment took into consideration both CD4 count (< or ≥200 cells per µL) and gender to result in a study population with approximately equal proportions. Subjects received monetary compensation ($160) for participation.

### Procedures

Oral rinse samples were collected every two weeks for six consecutive months (visits 1–14) using a 30 second Scope™ mouthwash rinse and gargle[5]. A self-administered questionnaire was completed at enrollment for measurement of baseline characteristics, however interval behavioral data were not ascertained. Venous blood was collected at visits one and 14 for measurement of CD4 count and HIV viral load. A Scope™ sample was collected per 20 rinse samples and processed similarly as a contamination control.

### Laboratory analysis

Oral exfoliated cells were separated by centrifugation, washed, and re-suspended with phosphate buffered saline and stored at −80°C.

DNA was purified by use of a modified protocol for Puregene DNA Purification (Gentra Systems, Minneapolis, MN)[5]. The presence of HPV genomic DNA of 37 HPV types (Low-risk types 6, 11, 38, 40, 42, 54, 55, 61, 62, 64, 67, 69, 70, 71, 72, 81, 83, 84, 89; High-risk types 16, 18, 26, 31, 33, 35, 39, 45, 51, 52, 53, 56, 58, 59, 66, 68, 73, 82[6]) was detected by PGMY09/11 L1 consensus primer PCR[7], and type-specified by hybridization to a prototype line blot (Roche Molecular Systems, Inc., Alameda, California). Samples positive for β-globin were evaluable, scored as negative or positive, and the HPV type(s) detected reported for positive samples.

HPV viral load for types 16, 18, 31, 33, and 35, chosen because of their association with OPSCC[8], was measured for all samples collected from individuals ever positive by line blot hybridization for the corresponding HPV type(s). Type-specific, Taq-Man, real-time PCR (Applied Biosystems, Foster City, CA) assays were performed as previously described[9], [10]. Each sample, including the standards, was assayed in duplicate, and a duplicate mean ≥ one copy of viral DNA was positive. To adjust oral HPV viral load to the number of oral epithelial cells present per PCR-reaction (see below), cell number was estimated by use of a TaqMan real-time PCR targeting a single copy human gene on chromosome 7, Human Endogenous Retrovirus 3 (ERV-3)[11], [12].

HIV viral load was measured by use of the Roche AMPLICOR™ HIV-1 Monitor Test, version 1.5 and reported from <400 to 750,000 copies per mL. CD4 cell counts were measured by use of the Beckton Dickenson BD TriTest CD4 FITC/CD8 PE/CD3 PerCP Reagent and reported as the number of CD4^+^ cells per microliter of blood (absolute count).

### Statistical analysis

Descriptive statistics were used to report demographic, biological and behavioral characteristics of the study population and to compare subjects who completed the study, defined as having attended visits one to 14 with a maximum of one interval missed visit, to those who were lost to follow-up. HPV infection status was determined at visits one through 14 and evaluated both in terms of individuals and for type-specific infections. In analysis by individual, a subject was considered infected if one or more of the 37 HPV types evaluated were detected at each visit (point prevalence) or detected in any of the samples collected over the course of the study (cumulative prevalence). In analysis by infection, presence of each of the 37 type-specific HPV infections was evaluated for each individual at each visit (point prevalence) or in any of the samples collected over the course of the study (cumulative prevalence).

Point and cumulative prevalence of oral HPV (and exact binomial 95% confidence intervals (CI)) were calculated for visits one through 14. Average point prevalence represented the weighted average of point prevalence for each visit, using the number of individuals or infections at a given visit as the weight. McNemar's test was used to compare the two-week and six-month cumulative prevalence estimates.

The primary goal was to evaluate HPV infection patterns over time, specifically to compare two-week to six-month sampling intervals. These analyses were restricted to the 91 individuals who completed the study. Under the six-month sampling interval, type-specific HPV infections were categorized as “persistent” (+/+) if present at both visit one and 14, “newly detected” (−/+) if absent at visit one and present at visit 14, “cleared” (+/−) if present at visit one and absent at visit 14 or “absent” (−/−) if not detected at either visits one or 14. These categories were distinct from those for the two-week sampling intervals in which infections were categorized as “persistent” if present in two or more consecutive visits, “transient” if detected without persistence, and “absent” if never detected. A persistent infection was considered “cleared” if not detected for at least two consecutive visits. An infection was defined as having “recurred” if it was detected at any visit after clearance.

To evaluate the relationship between HPV viral load and infection persistence, the adjusted HPV viral load for types 16, 18, 31, 33, and 35 was plotted across visits. The adjusted HPV viral load was the logarithmically converted ratio of (10^5^) HPV copy number (as measured by real-time PCR) divided by the number of cells in the sample (as measured by ERV-3 real-time PCR). A logistic transition model was used to evaluate the relationship between the magnitude of the adjusted viral load at one visit and a positive (i.e. non-zero) adjusted viral load at the next visit.

## Results

### Study population

The characteristics of the study population are shown in Table 1. Ninety-one of 112 (81%) individuals completed the six-month study. Of these, 74 completed all 14 visits and 17 missed a single visit. The median number of visits for the 21 individuals lost to follow-up was 10 (interquartile range: 4–12). There were no significant differences between patients who completed the study and those lost to follow-up (Table 1).

**Table 1:** Characteristics of the total study population and stratified by follow-up status.*

	Total N = 112	Completed V1-14 N = 91	Lost to Follow-up N = 21
**Age**			
Mean (SD)	45 (6.9)	44 (7.0)	46 (6.4)
Median (Range)	40 (28–61)	44 (28–60)	45 (37–61)
**Gender N (%)**			
Male	65 (58%)	54 (59%)	11 (52%)
Female	47 (42%)	37 (41%)	10 (48%)
**Race**			
Caucasian	8 (7%)	6 (7%)	2 (10%)
African	98 (88%)	79 (86%)	19 (90%)
American	6 (5%)	6 (7%)	-
Other	-	-	-
**Socioeconomic Status**			
<10K	65 (58%)	52 (57%)	13 (62%)
10–19K	17 (15%)	14 (15%)	3 (14%)
≥20K	12 (10%)	8 (9%)	4 (19%)
Unknown	18 (16%)	17 (19%)	1 (5%)
**Present Marijuana Use**			
Yes	30 (27%)	21 (23%)	9 (43%)
No	82 (73%)	70 (77%)	12 (57%)
**Present Tobacco Use**			
Yes	72 (64%)	55 (60%)	17 (81%)
No	39 (35%)	35 (39%)	4 (19%)
Unknown	1 (1%)	1 (1%)	-
**Heroin Use**			
Yes	61 (54%)	47 (52%)	14 (67%)
No	51 (46%)	44 (48%)	7 (33%)
**Enrollment CD4 Count**			
Mean (SD)	341 (275)	328 (237)	396 (403)
Median (Range)	282 (4.0–1251)	290 (4.0–1080)	200 (14–1251)
			
≥200	67 (60%)	57 (63%)	10 (48%)
<200	45 (40%)	34 (37%)	11 (52%)
**Enrollment HIV Viral Load**			
Undetectable (<400)	47 (42%)	39 (43%)	8 (38%)
400–20,000	25 (22%)	21 (23%)	4 (19%)
≥20,000	38 (34%)	29 (32%)	9 (43%)
Unknown	2 (2%)	2 (2%)	-

SD  =  standard deviation.

### HPV detection

In total, 1,434 of 1,568 (91%) scheduled visits were completed. Almost all (1,432 of 1,434 (99.9%)) oral rinse samples were β-globin positive. All controls for specimen collection and processing were appropriately negative for HPV and β-globin.

### Point and cumulative prevalence of oral HPV

**Figure 1:**
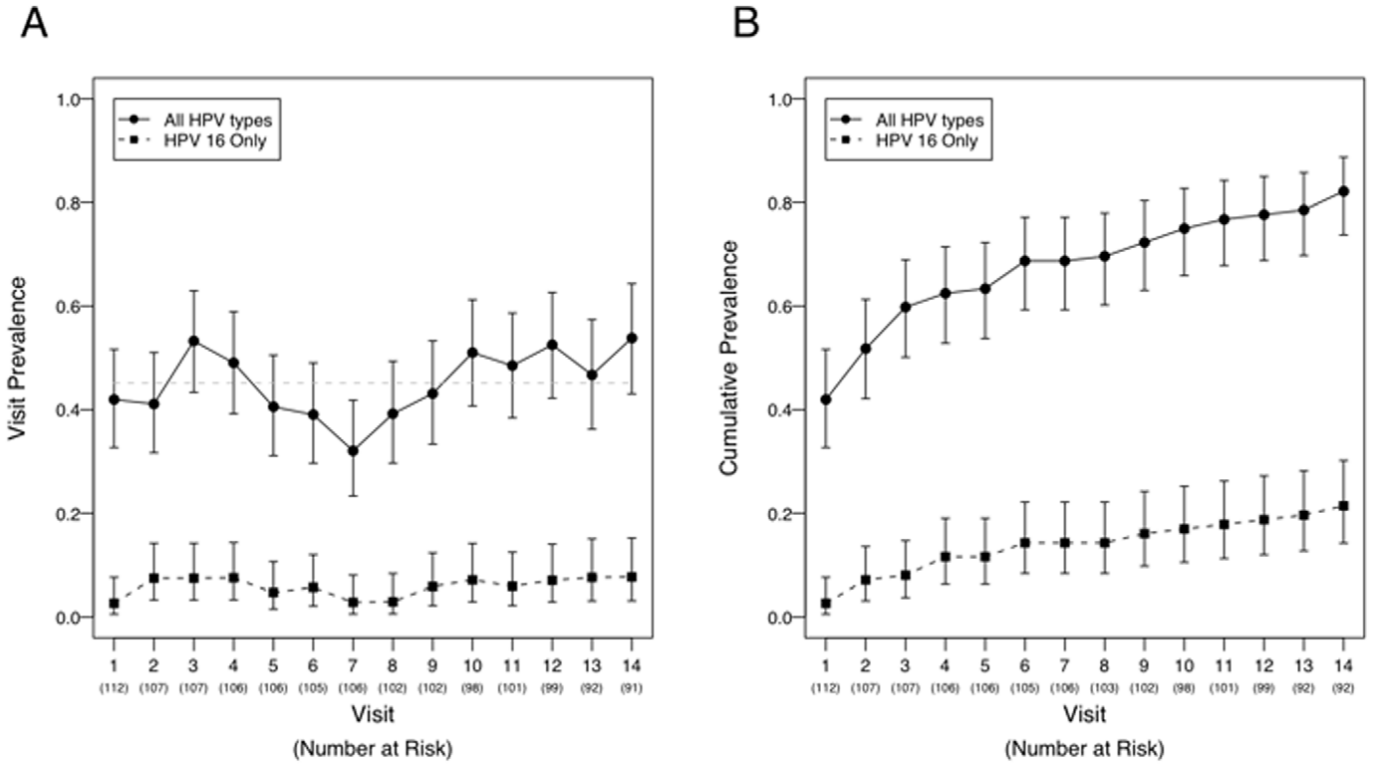
Point and cumulative prevalence of oral HPV infections in the study population. (a) The average point prevalence for oral HPV infections among individuals at visits 1 through 14 for all HPV types (solid line) and for HPV16 only (dashed line). A dotted grey horizontal line is drawn at 43%, the average point prevalence across visits. B) Cumulative prevalence inclusive of visits 1 through 14 for all HPV types (solid line) and for HPV16 only (dashed line).

First, we determined the proportion of the study population that had an oral HPV infection. The point prevalence for oral HPV infection measured at two-week sampling intervals remained relatively stable (range, 32–54%, Figure 1A). Average point prevalence for the two-week and six-month sampling intervals was similar (45%, versus 47%, *P* = 0.52) (Table 2 and Figure 1A). However, cumulative oral HPV prevalence, a measure of cumulative oral HPV exposure over the course of the study, was significantly higher with a two-week than a six-month sampling interval (82.1 versus 53%, *P*<0.0001, Figure 1B and Table 2).

**Table 2:** Average point and cumulative prevalence rates for oral HPV infection by individuals or type-specific infection in two-week or six-month sampling intervals.

	BY INDIVIDUAL						BY INFECTION					
	Average Point Prevalence			Cumulative Prevalence			Average Point Prevalence^1^			Cumulative Prevalence		
**Two-week Sampling**	**No. + visits for individuals/No. individual visits**	**%**	**95% CI**	**No. individuals + for at least one visit/No. individuals**	**%**	**95% CI**	**No. + test results/No. Tests**	**%**	**95% CI**	**No. Infections positive for at least one visit/No. infections tested**	**%**	**95% CI**
	646/1434	45.00	42.3, 47.6	92/112	82.10	73.7, 88.7	1549/53053	2.90	2.7, 3.1	386/4144	9.30	8.4, 10.2
**Six-month Sampling**	**No. + visits for individuals/No. individual visits**	**%**	**95% CI**	**No. individuals + for at least one visit/No. individuals**	**%**	**95% CI**	**No. positive test results/No. Tests**	**%**	**95% CI**	**No. Infections positive for at least one visit/No. infections tested**	**%**	**95% CI**
	96/203	47.30	40.2 54.4	59/112	52.70	43.0, 62.2	227/7509	3.00	2.6, 3.5	158/4144	3.80	3.2, 4.4

The point prevalence represents the average point prevalence at each visit weighted by the number of observations at each visit. Cumulative prevalence accounts for all individuals or infections that were HPV positive at visits 1 through 14 (two-week interval) or visits 1 through 14 (six-month interval).

1 A total of 5 individuals were missing a single HPV type for one visit.

CI  =  confidence interval.

We then evaluated type-specific oral HPV infections among the entire study population. We considered all possible infections, i.e. 37 infections from visits 1–14 (a total of 1, 434 evaluable samples) in 112 individuals (Table 2). The average point prevalence was similar for the two-week and six-month sampling intervals (3.0% versus 2.9%, *P* = 0.59). However, cumulative prevalence of detection of 37 type-specific infections was significantly higher with a two-week than a six-month sampling interval (9.3% (n = 386 infections) versus 3.8 (n = 158 infections), *P*<0.0001). The average point prevalence for each of the 37 type-specific infections ranged from 2.1–3.9% at the two-week sampling intervals (Table 3, data only shown for HPV types 16, 18, 31, 33, 35).

**Table 3:** The average point prevalence, cumulative prevalence and percentage of all detected infections in visits 1 through 14, stratified by HPV risk category.

	AVERAGE POINT PREVALENCE			CUMULATIVE PREVALENCE			PROPORTION OF ALL INFECTIONS		
	No. + Tests/No. Tests	%	95% CI	No. infections + for at least one visit/No. infections tested	%	95% CI	No. HPV Type X /No. HPV infections detected	%	95% CI
**High Risk HPV Types**	878/25811	3.4	3.1, 3.6	219/2016	10.9	9.5, 12.3	219/386	56.7	51.6, 61.8
**HPV 16**	84/1434	5.9	4.6, 7.2	24/112	21.4	14.2, 30.2	24/386	6.2	4.0, 9.1
**HPV 18**	38/1434	2.6	1.8, 3.6	23/112	20.5	13.4, 29.2	23/386	6.0	3.8, 8.8
**HPV 31**	10/1434	0.7	0.3, 1.3	4/112	3.6	0.9, 8.9	4/386	1.0	0.2, 2.6
**HPV 33**	22/1434	1.5	0.9, 2.3	7/112	6.3	2.5, 12.5	7/386	1.8	0.7, 3.7
**HPV 35**	92/1434	6.4	5.2, 7.8	23/112	20.5	13.4, 29.2	23/386	6.0	3.8, 8.8
**Low Risk HPV Types**	671/27242	2.5	2.2, 2.7	167/2128	7.8	6.7, 9.1	167/386	43.3	38.2, 48.4

For HPV16 infection, the average point prevalence was 5.9% (95% CI: 4.6, 7.2) and the cumulative prevalence was 21.4% (95% CI: 14.2, 30.2). HPV infections with the highest point prevalence (pp) and cumulative prevalence (cp) were HPV39 (pp = 12%, cp = 28%), HPV52 (pp = 8%, cp = 21%), HPV35 (pp = 6%, cp = 21%) and HPV16 (pp = 6%, cp = 21%).

### Patterns of HPV infection

**Figure 2:**
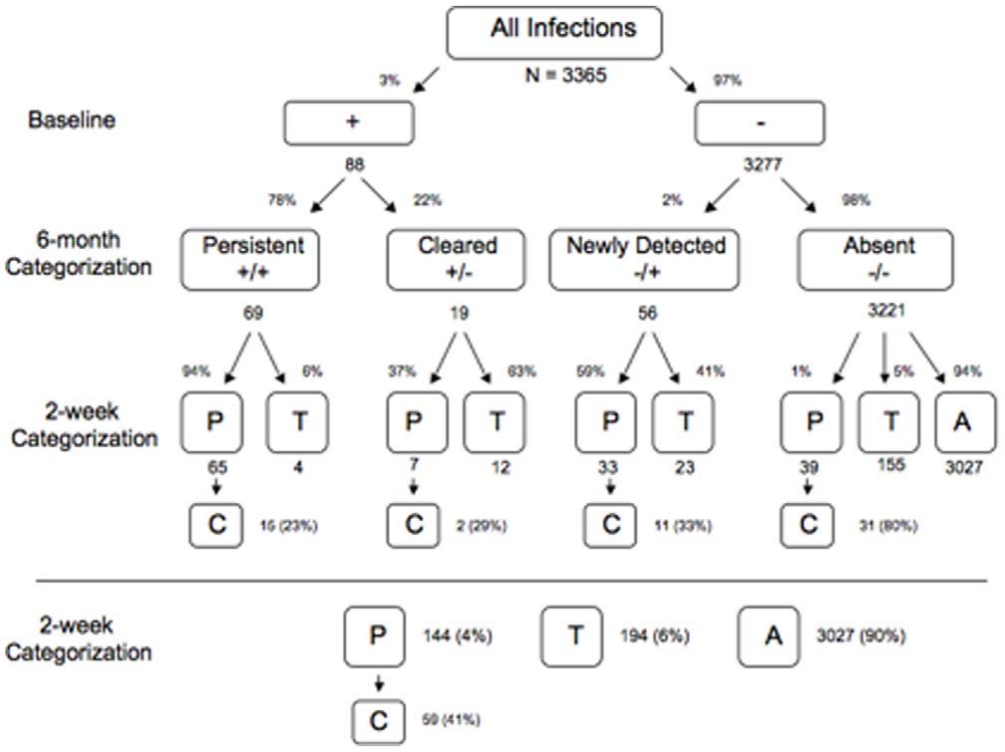
The relationship between type-specific oral HPV infections detected at two-week and six-month sampling intervals. The flow chart shows the relationship between oral HPV infections detected at a six-month sampling interval (visits 1 and 14, above the horizontal line) and categorized as persistent (+/+), cleared (+/−), newly detected (−/+), or absent (−/−) and their categorization as persistent (P), transient (T), or absent (A) infections under the two-week interval definitions (see methods). Persistent infections under the two-week categorization that subsequently cleared (C) are also indicated. However, cleared infections which recurred are not included in the figure, refer to text. A summary of infections as categorized under a two-week sampling interval as persistent, transient, absent (A) and cleared is included below the horizontal line. A total of 3365 possible infections were evaluated at visits 1 and 14 ((91×37) −2).

The patterns of HPV infection were evaluated for both two-week and six-month sampling intervals among the 91 individuals who completed visits 1 and 14 (Figure 2). Among these 91 individuals, we evaluated a total of 3,365 possible infections, i.e. 37 HPV types among 91 individuals, minus two samples missing data on one HPV type or (37×91) −2 = 3,365.

#### Six-month sampling results

At visit one, 88 HPV infections were detected in 41 of 91 (45%) participants. At the final visit, 69 (78%) were categorized as persistent and 19 (22%) were categorized as cleared (Figure 2), and 56 infections were newly detected. The remaining 3,221 infections (96% of all possible infections) were absent at visit one and 14 (Figure 2).

#### Two-week sampling results

As would be expected, the number of infections detected with a two-week sampling interval was higher than for a six-month sampling interval (Figure 2). In total, 338 of 3,365 (10%) possible infections were detected. By the two-week sampling definition, 194 were transient, 144 were persistent, and 59 (41%) of these persistent infections were categorized as cleared. Of the infections that cleared, 42 (71%) recurred, 27 (46%) of which again met criteria for persistence. The median percentage of visits an infection was detectable was 14% (range, 7–100%), and 83 infections (25%, CI: 20–30%) were detectable for the majority (>50%) of visits. Only 24 of 144 persistent infections under a two-week definition were detectable at all subject visits.

#### Comparison of sampling schemas

We then compared the HPV detection results obtained with a two-week to a six-month sampling interval (Figure 2). Almost all (65 of 69, 94%) infections categorized as persistent under the six-month definition were persistent under the two-week definition. Similarly, almost all (3,027 of 3,221, 94%) infections categorized as absent under the six-month definition were also absent under the two-week definition. The majority of the 194 additional infections detected with a two-week sampling interval were transient.

**Figure 3:**
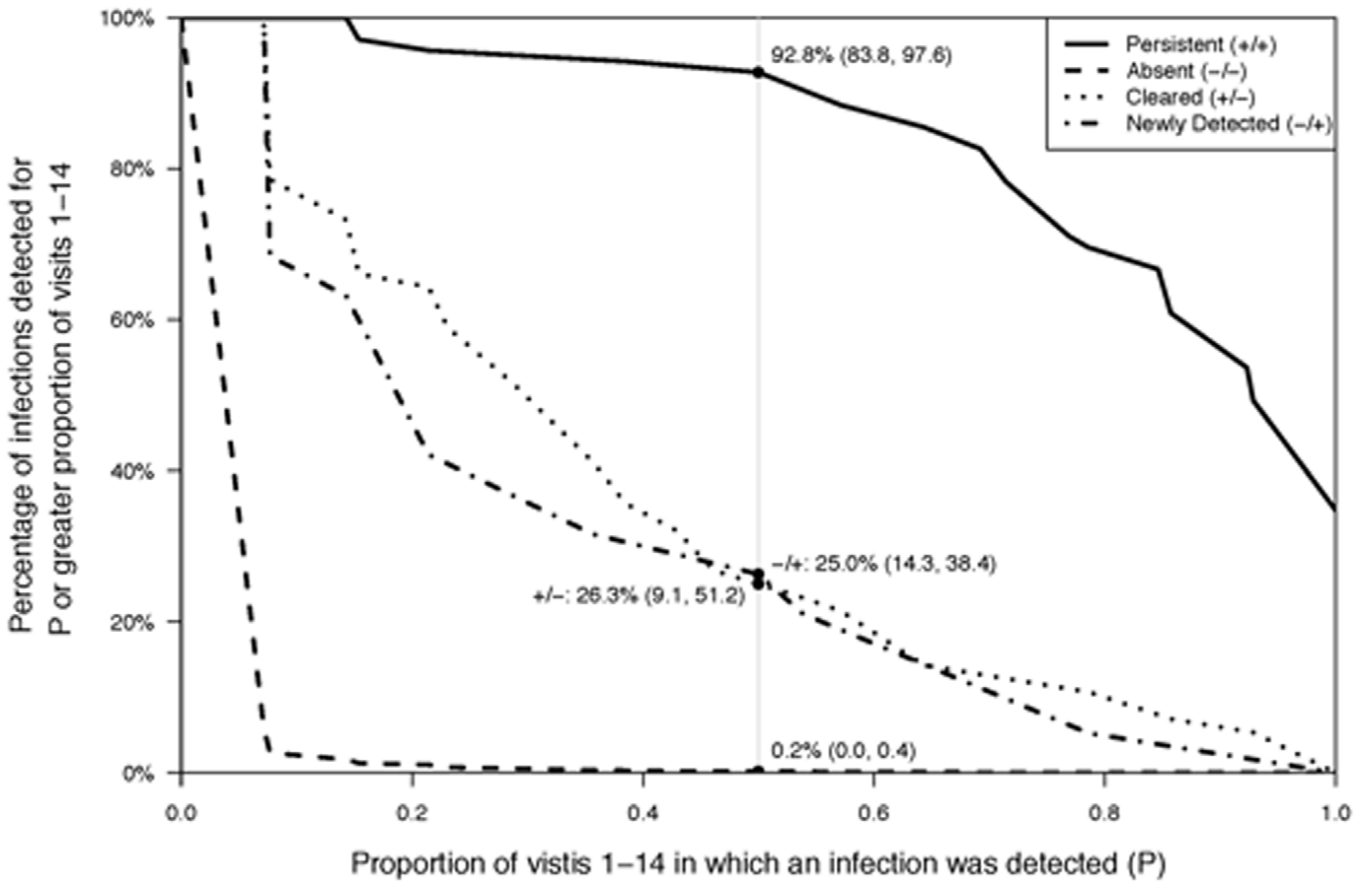
Probability of type-specific oral HPV infection detection depending upon the proportion of the two-week visits that an infection was detected, stratified by six-month categorization as persistent, absent, cleared or newly detected infection. For each six month interval categorization of oral HPV type-specific infection status (+/+, −/−, +/−, −/+), the probability of observing a positive test result for a fixed proportion of two week interval visits or greater is plotted as a function of the proportion of two-week interval visits (P). The vertical grey line highlights the case in which at least half of the visits are positive, P = 0.5 (see results text).

We evaluated the probability that an infection was detectable for a certain percentage of the two-week visits stratified by their categorization under the six-month sampling interval as persistent, cleared, newly detected or absent (Figure 3). Oral HPV infections defined as persistent under the six-month sampling interval had a high probability (0.93, 95% CI: 0.83, 0.98) of being detected at more than 50% of the two-week visits. Similarly, there was very low probability (0.002, 95% CI: 0.00, 0.004) that oral HPV infections that were absent at both visits 1 and 14 were detected at more than 50% of intervening visits. Indeed, if a type-specific HPV infection was not detected at either visit 1 or 14, the probability that the infection was not detected with a two-week sampling interval was 0.94 (95% CI: 0.93, 0.95). Probabilities for cleared and newly detected infections were largely indistinguishable. The likelihood that a cleared or newly detected infection by the six-month sampling interval definition was detectable at 50% or more of the two-week visits was equivalent (0.26 and 0.25, respectively).

### Factors affecting persistence

**Figure 4:**
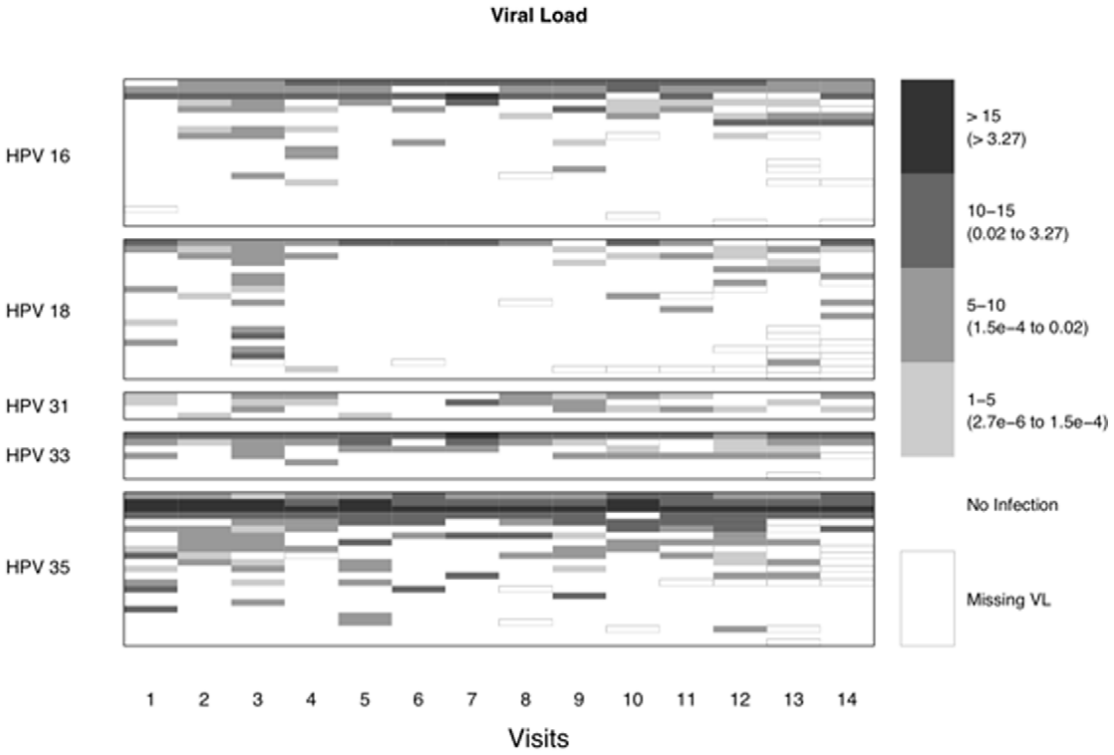
Patterns of adjusted HPV viral load (VL) at two-week intervals for individuals positive by line blot for HPV types 16, 18, 31, 33, and 35. White cells and grey cells represent the absence and presence, respectively, of an HPV infection as determined by real-time PCR. The grey scale indicates ordinal categories of increasing adjusted HPV VL values (from light gray to dark gray). The adjusted HPV VL represents the log of 10^6^ times the ratio of HPV copies to ERV-3 copies. Missing VL values are indicated by outlined cells. The blank lines represent 11 infections detected by line blot assay that were either not detected (10) or had missing data (1) by VL.

We evaluated the relationship between HPV viral load and persistence or clearance of five high-risk HPV types (HPV16, 18, 31, 33, or 35). The pattern of viral load over the course of the study for those individuals with at least one positive line blot assay for a specific high-risk HPV type is shown in Figure 4. Upon visual inspection, infections present across multiple visits appeared to have a higher viral load (Figure 4, darker intensity bars) than those inconsistently present over time (Figure 4, lighter intensity bars). This visual impression was statistically confirmed in a transition analysis that evaluated whether the odds of a positive oral HPV test at a given visit was affected by the HPV viral load at the prior visit (Table 4). For each HPV type evaluated, the odds of a positive test were significantly increased among individuals who tested positive at the previous visit. Additionally, there was a significant increase in the odds of a HPV-positive test for each unit increase in adjusted HPV copy number from the previous visit (OR range for HPV 16, 18, 33, 35: 1.26–1.51, *P*-values range: <0.0001–0.0001). Similar transition analyses focusing on line blot analysis results for the six-month interval sampling (visits 1 and 14) and baseline characteristics (age, gender, smoking, CD4 count, viral load, and HPV serostatus) did not show any significant associations (data not shown).

**Table 4:** Odds of detection of a type-specific HPV infection as a function of adjusted HPV viral load (via RT-PCR) at the prior visit.

	+ Given Previous −			+ Given Previous +			e^β^	P-value	e^δ^	P-value
	(+/−)	%	95% CI	(+/+)	%	95% CI				
HPV 16	24/209	11	7, 17	45/70	64	51, 76	14.45	<0.0001	1.51	<0.0001
HPV 18	29/209	14	9, 20	18/46	39	25, 55	3.99	0.0001	1.26	0.0001
HPV 31	12/32	38	21, 57	11/21	52	29, 75	1.83	0.2870	1.29	0.1310
HPV 33	11/53	21	10, 35	30/39	76	59, 89	12.30	<0.0001	1.44	<0.0001
HPV 35	34/172	20	14, 27	83/117	71	61, 79	9.91	<0.0001	1.33	<0.0001

**e** ^β^represents the ratio of the odds of an infection among individuals with and without an infection at the previous visit.

**e** ^δ^represents the change in the odds of infection per unit increase of adjusted viral load at the previous visit.

## Discussion

Our data indicate a very low probability of interval detection of a type-specific oral HPV infection if the type was not detected at a six-month sampling interval. Furthermore, there was a very high probability that a type-specific infection categorized as persistent at a six-month sampling interval was detectable for the majority of the intervening two-week visits. A six-month sampling interval therefore appears appropriate for natural history studies of oral HPV infection that model factors associated with type-specific persistence.

Cervical HPV natural history studies serve as a model for development of analogous studies at other anatomic sites (e.g. oral and anal). The clinical algorithm for cervical cancer screening predated natural history studies of cervical HPV infection and predetermined the commonly used six-month sampling interval. Shorter sampling intervals were not examined prior to implementation of large, well-designed landmark natural history studies. The expense, complexity and burden of serial anogenital sampling likely prohibited more frequent sampling. Consequently, cervical infections that would be detected at a shorter interval are generally believed to not be pathologically significant, while persistence of infection at six-month intervals significantly elevates risk for cervical dysplasia[13] and thus has been established as a surrogate for this disease outcome [14]. Given the growing recognition of oral HPV infection as a risk factor for oral cancers, we elected to examine a priori the potential biases introduced by sampling interval, rather than to assume the cervical standard of a six-month sampling interval.

Our initial studies of the natural history of oral HPV infection have focused on a study population with high prevalence, HIV-infected individuals. We acknowledge that the behavior of infections in this population likely differs from what would be observed in an immunocompetent population. For example, transient detection of oral HPV infection was commonly observed with two-week sampling, and the underlying reasons for this observation are unclear. This may be attributable to technical issues, such as variability in factors affecting oral rinse sampling over time, or fluctuation of HPV viral load above and below the lower limit of detection of the Roche line blot, which is known to vary by type. Consistent with such fluctuation around the limit of assay sensitivity is our finding that the majority of cleared infections under the two-week definition subsequently recurred and met criteria for persistence. Although laboratory contamination of the specimen could also be an explanation, we did not observe any evidence of contamination in control samples. Alternatively, in an immunosuppressed population the high frequency of transient, low viral load infection may represent intermittent reactivation of infections due to immunosuppression. Transient detection may also represent exposure in the absence of subsequent establishment of infection. We did not have the interval sexual behavior data which may have allowed us to explore this possibility.

The biological significance of transient HPV infections in the oral cavity, oropharynxand cervix is unclear. While the majority of cervical HPV infections are transient infections, persistent infections are significantly more likely to progress to both premalignant and malignant lesions[13]. However, even transient cervical HPV infections (defined as an HPV infection detected at only one visit within a 3 to 12 month period) have been associated with an approximate 5-fold (OR 5.5 95%CI 1.4–21.9) increased risk of squamous intraepithelial lesions in comparison to no detection[15]. High HPV viral load is also associated with elevated risk for incident cervical intraepithelial neoplasia 2 (CIN II) or more invasive pathology[16]. Our two-week interval analysis suggests that infections with a high HPV viral load were more likely to persist than low viral load infections, but an important limitation to this study is that associations with disease outcomes in the oral cavity were not possible.

Our data lend support to the use of a six-month sampling interval for studies designed to evaluate factors associated with type-specific infection persistence. However, misclassification of transient oral HPV infections as absent would result in an overestimation of the median time to clearance of incident oral HPV infections. A further limitation is that HPV viral load was measured only among individuals with at least one positive line blot assay of the corresponding HPV type. Low copy number oral HPV infections below the lower limit of sensitivity of the line blot would not be selected. As these low copy number infections would be expected to have a higher clearance rate than high copy number HPV infection, we may have underestimate the association between viral load and persistence.

Oral HPV prevalence in the study population appeared to be a reasonable approximation of the proportion infected over time. However, it is important to note the potential effect of missing data, which may have caused us to underestimate cumulative prevalence. The difference in cumulative prevalence estimates at two-week and six-month sampling intervals indicate that a six-month interval sampling may significantly underestimate the proportion of the study population that has been “exposed” and misclassify individuals as “unexposed”. Therefore, a six-month sampling interval may bias point estimates for factors that increase risk of acquiring an oral HPV infection toward the null.

Despite these limitations, this is the first short term natural history study of oral HPV infection and the first to report results on more than two consecutive visits. Based upon this data, a six-month sampling interval is currently being used in our natural history studies of oral HPV infection to evaluate factors affecting type-specific oral HPV persistence.
